# Eukaryotic initiation factor 4F promotes a reorientation of eukaryotic initiation factor 3 binding on the 5′ and the 3′ UTRs of barley yellow dwarf virus mRNA

**DOI:** 10.1093/nar/gkac284

**Published:** 2022-04-21

**Authors:** Paul Powell, Usha Bhardwaj, Dixie Goss

**Affiliations:** Department of Chemistry, Hunter College, CUNY, New York, NY 10065, USA; Ph.D. Program in Chemistry, The Graduate Center of the City University of New York, New York, NY 10016, USA; Department of Chemistry, Hunter College, CUNY, New York, NY 10065, USA; Department of Chemistry, Hunter College, CUNY, New York, NY 10065, USA; Ph.D. Program in Chemistry, The Graduate Center of the City University of New York, New York, NY 10016, USA; Ph.D. Program in Biochemistry, The Graduate Center of the City University of New York, New York, NY 10016, USA

## Abstract

Viral mRNAs that lack a 5′ m^7^GTP cap and a 3′ poly-A tail rely on structural elements in their untranslated regions (UTRs) to form unique RNA-protein complexes that regulate viral translation. Recent studies of the barley yellow dwarf virus (BYDV) have revealed eukaryotic initiation factor 3 (eIF3) plays a significant role in facilitating communication between its 5′ and 3′ UTRs by binding both UTRs simultaneously. This report uses *in vitro* translation assays, fluorescence anisotropy binding assays, and selective 2′-hydroxyl acylation analyzed by primer extension (SHAPE) footprinting to identify secondary structures that are selectively interacting with eIF3. SHAPE data also show that eIF3 alters its interaction with BYDV structures when another factor crucial for BYDV translation, eIF4F, is introduced by the 3′ BYDV translational enhancer (BTE). The observed BTE and eIF4F-induced shift of eIF3 position on the 5’ UTR and the translational effects of altering eIF3-binding structures (SLC and SLII) support a new model for BYDV translation initiation that requires the reorientation of eIF3 on BYDV UTRs. This eIF3 function in BYDV translation initiation is both reminiscent of and distinct from eIF3–RNA interactions found in other non-canonically translating mRNAs (e.g. HCV). This characterization of a new role in translation initiation expands the known functionality of eIF3 and may be broadly applicable to other non-canonically translating mRNAs.

## INTRODUCTION

Most RNA viruses have unique translation mechanisms that utilize only a subset of the eukaryotic translation initiation factors (eIFs) required for canonical translation of cellular mRNAs. Many RNA viruses lack the post-transcriptional modifications of a 5′ 7-methylguanosine cap and a 3′ poly-adenosine (poly-A) tail. Because of this, two eIFs frequently bypassed in viral translation mechanisms are the cap-binding initiation factor 4E (eIF4E) and the poly-A binding protein (PABP) (1).

Cellular mRNAs undergoing canonical translation use eIF4F (a complex of eIF4E and eIF4G in plants) and PABP to bridge the two ends of the mRNA. This eIF4F-PABP bridge facilitates communication between the UTRs and serves as the basis of the closed-loop model of canonical translation. Several important functions have been ascribed to this canonical 5′-3′ mRNA cyclization including promotion of ribosome recycling and prevention of mRNA decay which both ultimately enhance translation efficiency (2–4). This allows cells to alter poly-A tail length or clip off the 5′ cap to promote mRNA degradation and regulate gene expression (5).

RNA viruses that lack a 5′ cap and 3′ tail can garner similar degradation-preventing and translation-enhancing benefits by using structural elements in their untranslated regions (UTRs) that form direct RNA-RNA interactions between UTRs and that recruit eIF4F or other eIFs to facilitate bridging between UTRs. These UTR-bridging complexes may also serve functions unique to RNA viruses like toggling between translation and replication (6,7). Many RNA plant viruses utilize cap-independent translational enhancers (CITEs), which are RNA structures usually found in the 3′ UTR. CITES recruit important factors like eIF4F or the 40S ribosomal subunit which must either subsequently or simultaneously interact with the 5′ UTR for translation to proceed (8).

**Figure 1.**
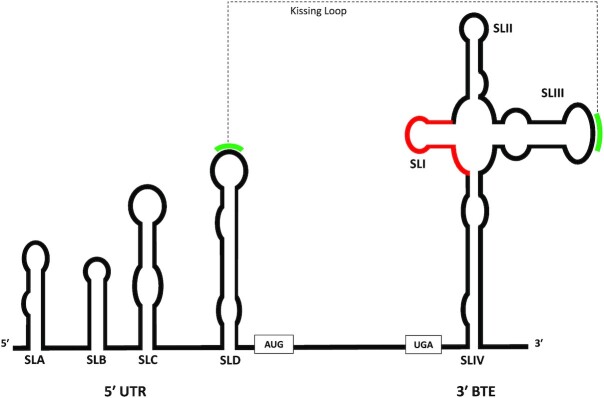
The BYDV UTR structures involved in translation initiation. The 5′ UTR comprises four stem loops (SLA-SLD) while the 3′ UTR contains the cruciform BTE structure containing its own four stem loops (SLI-SLIV). The complementary loops (SLD and SLIV) involved in the translationally required long distance RNA-RNA kissing loop interaction are marked in green. The 17-nt conserved sequence in BTE is marked in red.

Barley yellow dwarf virus (BYDV, Genus *Luteovirus*) contains a BTE (BYDV-like CITE) structure in its 3′ UTR. BTE structures are found in all members of the *Luteovirus*, *Dianthovirus* and *Necrovirus* genera and in some umbraviruses. All BTE structures contain a conserved 17 nucleotide sequence that binds eIF4F (via eIF4G) and forms SLI, the first of up to six stem–loops that all extend from a central hub. In most BTEs one stem–loop contains a hairpin sequence that is complementary to a hairpin in the 5′ UTR (8). In BYDV the BTE-SLIII hairpin contains a five-base sequence that is complementary to the hairpin of stem–loop D (SLD) in the 5′ UTR (Figure 1). This long-distance, kissing-loop interaction and the recruitment of eIF4F to the 3′ BTE structure are both required for BYDV translation (9–11). These translational requirements are partially explained by the recruitment of the 40S ribosome onto BTE that happens in the presence of an active helicase complex (eIF4A, eIF4B, eIF4F and ATP) (12). However, that helicase complex alone is unable to facilitate 40S binding to BYDV 5′ UTR nor can it produce 40S toeprints at the start codon even in the presence of BTE unless additional factors are present. Initiation factor 3 (eIF3) has been identified as one factor that, in conjunction with the active helicase complex, bridges the 5′ and 3′ UTRs of BYDV and allows 40S to bind the BYDV 5′ UTR. Interestingly, this eIF3-helicase complex can generate 40S binding to the 5′ UTR even in the absence of eIF4F and BTE (13). The existence of a BTE- and eIF4F-independent means of 40S-5′ UTR binding suggests that further exploration of the BTE-bound eIF4F influence on a 5′-bound complex could reveal new mechanistic details of BYDV translation initiation.

In this report, *in vitro* translation assays and anisotropy binding are used to show physiological relevance of eIF3-UTR interactions, and selective 2′-hydroxyl acylation analyzed by primer extension (SHAPE) footprinting of the BYDV UTRs in the presence of mixtures of initiation factors (14) is used to determine how the presence or absence of both BTE and eIF4F affects eIF3-UTR interactions. Together eIF4F and BTE induce a shift of the eIF3 binding sites on BYDV RNA. The eIF3-responsive nucleotides are highlighted in tertiary structural predictions of BYDV RNA structures and incorporated into an updated model of BYDV translation initiation. The functions of eIF3 in our updated model of BYDV translation initiation are distinct from, but reminiscent of roles eIF3 has in HCV-like viral translation (15,16) and in non-canonically translating cellular mRNAs like the XIAP internal ribosomal entry site (IRES) (17). This characterization of a novel eIF3 role in translation initiation expands the known repertoire of eIF3 functionality and may be broadly applicable for characterization of eIF3 activity in many non-canonically translating mRNAs.

## MATERIALS AND METHODS

### Purification of initiation factors

Recombinant wheat eIF4A, eIF4B and eIF4F were prepared as described previously (18,19) using clones generously provided by Dr. Daniel Gallie (University of California, Riverside, CA, USA) and Dr Karen Browning (University of Texas at Austin, Austin, TX, USA). Native eIF3 protein was purified from wheat germ extract as reported previously (13).

### RNA preparation and design

**Figure 2.**
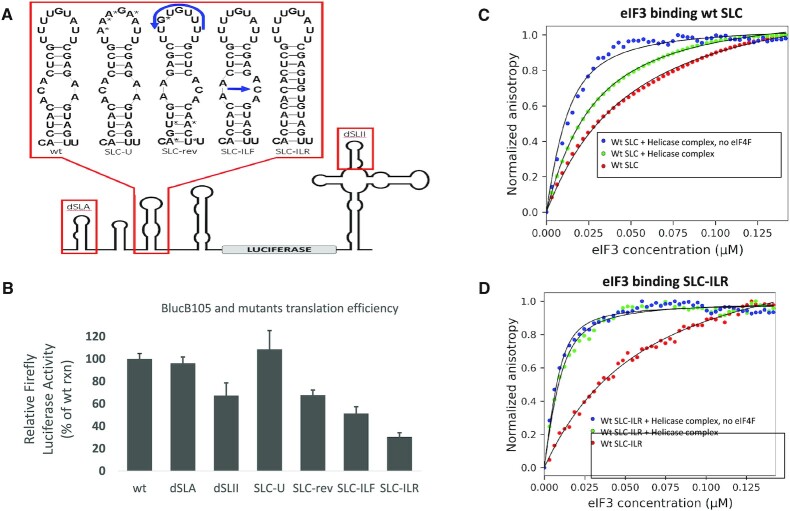
(**A**) Mutations of the BlucB105 reporter construct comprise two deletion mutants and four alterations of SLC. The dSLA mutant deleted SLA from 5′ UTR. The dSLII mutant removed SLII from 3′ BTE. The SLC-U mutant replaced four uracils in the 5′-SLC hairpin with Adenines. The modified nucleotides are marked with asterisks. The SLC-rev mutants reversed the full SLC sequence except for two unaltered base pairs and one point mutation in the hairpin (marked with asterisks) that removed start codons. The SLC-ILF mutant flips the 5′ and 3′ sides of the SLC internal loop (SLC-ILF), and the SLC-ILR mutant fully removes the SLC internal loop. (**B**) Relative luciferase luminescence intensity shows that dSLII and SLC mutants that altered the internal loop (-rev, -ILF, and -ILR) decreased translation efficiency. Plots (**C**) and (**D**) show fluorescence anisotropy binding of eIF3 to wt SLC and SLC-ILR, respectively. Plots similar to that depicted in Panel D were obtained for the other mutants (Table 1).

Four mutants of the 5′ UTR stem–loop C (SLC) were designed for use in anisotropy binding studies and *in vitro* translation (IVT) assays. The sequences of SLC-U, SLC-rev, SLC-ILF and SLC-ILR mutants used in IVT and binding studies are shown in Figures 2A and Supplementary Table S1, respectively. SLC-U exchanges four uracils for adenines in the U-rich hairpin of SLC. SLC-rev reverses the entire SLC sequence. Additional point mutations were added to SLC-rev for use in IVT to avoid the introduction of uORFs. SLC-ILF flips the 5′ and 3′ sides of the SLC internal loop. SLC-ILR removes the internal loop by making the 3′ side of the loop complementary to the 5′ side. Isolated 5′-Fluorescein labeled SLC RNA and three SLC RNA mutants (SLC-U, SLC-rev and SLC-ILR) were purchased from IDT and used directly in anisotropy binding assays. Isolated SLC and SLC mutant oligomers used for anisotropy binding studies contain two additional, terminal GC base pairs to increase loop stability during binding.

BlucB_105_ is a reporter plasmid used for IVT assays that contains the firefly luciferase gene flanked by the BYDV genomic 5′ UTR and 3′ BTE (105 nt). This reporter is a truncated version of BlucB (originally designated LUC869) (20) which contained the full BYDV 3′ UTR. Mutants of BlucB_105_ were made replacing SLC with each of the four SLC mutants explained previously (excluding the added GC pairs) and two deletion mutants that removed either SLA from the 5′ UTR (dSLA) or SLII from BTE (dSLII). These mutants were prepared using the Q5 Site-Directed Mutagenesis Kit (NEB). The BlucB_105_ Plasmid and its mutants were linearized using SmaI restriction enzyme (NEB), transcribed using the HiScribe T7 High Yield RNA Synthesis Kit (NEB), and purified using the ssDNA/RNA Clean & Concentrator kit (Zymo Research).

Two RNA oligomers were designed for use in SHAPE and SHAPE footprinting studies (Supplementary Table S1). To study 5′ UTR by itself a template for the first 220-nt of the BlucB reporter plasmid was PCR amplified using the forward primer TAATACGACTCACTATAGCTC and reverse primer CAGTTGCTCTCCAGC (IDT). This included the full 5′ UTR and a piece of the luciferase mRNA. The Luciferase extension served as an annealing site for the reverse transcription primer used in SHAPE analysis. The RT primer has the same sequence as the reverse PCR primer plus a 5′-cyanine 5 label. The fusion (FUS) RNA oligomer from 5′ to 3′ ends contained the BYDV 5′ UTR, a 12-nt linker sequence, the BYDV BTE, and a piece of luciferase mRNA complementary to the same RT primer used for SHAPE analysis of the 5′ UTR. The FUS RNA was generated from a synthetic DNA template with a T-7 promoter sequence ordered from IDT.

Transcription reactions and purification of short RNA oligomers followed the same procedure described for BlucB_105_ reporter RNAs, excluding plasmid linearization. All RNA concentrations were determined using a Thermo Scientific NanoDrop 1000 spectrometer and RNA integrity was verified by 1% agarose gel electrophoresis. Some of the purified FUS RNA was 3′-fluorescein labeled (21) and used in anisotropy binding assays. All RNA was refolded as described under SHAPE footprinting experiments.

### Fluorescence anisotropy binding experiments

Fluorescence anisotropy experiments were performed to assess binding of 5′ SLC and FUS RNA oligomers to eIF3 in the presence and absence of the helicase factors (eIF4A, eIF4B, eIF4F and ATP). ATP sodium salt was used for most experiments, however binding experiments using ATP-MgCl_2_ showed no difference in binding of the factors. This complex is involved in BYDV translation initiation and enhancement of eIF3-BYDV binding. All fluorescence anisotropy binding experiments were performed and analyzed as described previously (13) except that 10nM RNA or oligonucleotide solutions were used. Anisotropy binding figures were generated using Kintek Explorer software (22,23).

### Luciferase-based translation efficiency assays

The BlucB_105_ reporter gene contains a firefly luciferase gene surrounded by the full BYDV 5′ UTR and the 105 bases of 3′ BTE (9). IVT assays were performed by adding 1 μg of wt BlucB_105_ or mutant BlucB_105_ RNA to 25 μl *in vitro* reaction mixtures made using the wheat germ extract (WGE) *in vitro* translation system from Promega. The reaction mixtures contained 50% (v/v) WGE (Promega), 80 μM amino acid mixture (Promega), 60 mM potassium acetate (Promega), and 1.6 units/μl RiboLock RNase inhibitor (ThermoScientific). After addition of RNA to the mixtures, reactions were incubated at 25°C for 40 min and stopped by the addition of 60 μM puromycin. 3 μl of each reaction was added to 30 μl Bright-Glo luciferase assay reagent (Promega) and measured for luminescence using a SpectraMax iD5 microplate reader (Molecular Devices) with default endpoint luminescence settings. Each reaction was measured in triplicate and repeated at least 5 times. Relative translational efficiency of each RNA was determined in relation to wt BlucB_105_ reacting concurrently and using WGE from the same batch. Averages and standard deviations of all translation reactions were calculated and graphed in excel.

### SHAPE and SHAPE footprinting

SHAPE footprinting reactions followed established protocols with some modifications (24–26). Briefly, RNA was refolded in 1X SHAPE buffer (20 mM HEPES, 300 mM KCl, 2 mM DTT, 5 mM MgCl_2_, pH 7.5) by incubation for 5 min at 65°C followed by 1 min on ice. 10 pmol of refolded RNA were added to 25 μl reactions containing 1× SHAPE buffer, 1 μl RiboLock RNase inhibitor (ThermoScientific) and eIF mixtures (0.3 μM each of eIF4A, eIF4B, eIF4F, 0.25 μM eIF3 and 5 mM ATP). eIF mixtures without eIF4F, eIF3, or both were also prepared. RNA was incubated with the initiation factor mix for 30 min at 37°C, then reacted with 3 μl of 14 mM (25 mM for larger FUS RNA) 1-methy-7-nitroisatoic anhydride (1M7) (27) for 70 s. 1M7 modified RNA was purified using ZYMO clean and concentrator spin columns with 2× 800 μl washes with DNA/RNA wash buffer and final elution in 10 μl DNase/RNase-free water. Negative controls were prepared in the same manner without addition of any eIFs and with 3 μl DMSO in place of 1M7. The Purified RNA was brought to 19 μl by adding 10 pmols of 5′-cyanine5 labeled cDNA SHAPE primer /Cy5/CAGTTGCTCTCCAGCGG (IDT), 1 μl RiboLock, 2 μl of 10 mM dNTP mix (NEB), 1 μl DTT 0.1 mM and 4 μl 5× first-strand buffer (Invitrogen). This mixture was heated to 65°C for 5 min and allowed to slowly cool to 50°C for primer annealing. Once at 50°C, 1 μl of SuperScript III reverse transcriptase (Invitrogen) was added and allowed to react for 40 min. Sequencing data was produced by running additional RT reactions on unmodified RNAs in the presence of ddNTPs. Final cDNA was recovered by adding 2 μl 2M NaOH at 95°C for 3 min to digest the RNA, neutralizing with 2 μl 2M HCl, adding 3 μl 3M sodium acetate as a precipitant, adding 80 μl 100% EtOH and pelleting the cDNA. Pellets were dried and resuspended in 60 μl SLS buffer (Sciex). 2 μl of each resuspended pellet was added to a 96 well plates for analysis, diluted to 40 μl SLS and mixed with 0.5 μl 400 bp DNA Size Standard (Sciex). Final cDNA was analyzed by capillary electrophoresis (CE) using a Beckman GenomeLab GeXP Genetic Analysis System using custom CE parameters optimized for footprinting analysis (28). CE data was aligned using MATLAB CEQ alignment software from the Laedearch Lab (UNC Chapel Hill) and analyzed using the SHAPEfinder software from the Giddings Lab (UNC Chapel Hill) (29). SHAPEfinder results for different eIF mixtures were compared against each other in excel.

To characterize eIF-RNA interactions on the 5′ and 3′ UTRs, SHAPE footprints (14,30,31) were collected for both the 5′ UTR and FUS oligomer in the presence of several combinations of initiation factors. All footprinting experiments included the active helicase factors eIF4A, eIF4B and 5 mM ATP (ATP sodium salt from Thermofisher) which were the minimum requirement for eIF3 binding to individual BYDV UTRs (13). We did not observe a significant difference when ATP-MgCl_2_ was used, however Mg^2+^ will differ in experiments where ATP sodium salt was used depending on the Mg^2+^ partitioning between the RNA and ATP. Helicase factors eIF4A and eIF4B alone do not have strong affinity for either 5′ or 3′ UTR, and eIF4F only shows strong affinity for the 3′ BTE (19). To correct for the effect of any weak or transient interactions between helicase factors and RNA, footprints of the eIF3 complexes in the presence of helicase factors were compared against footprints of helicase factor mixtures without eIF3. Each combination of initiation factors and RNA was analyzed three times and each result was measured against three, factor-free negative controls (*n* = 9). The mean differences between sets of reaction conditions were calculated and subject to a two-sided Student *t*-test to show which nucleotides had statistically significant changes in SHAPE reactivity caused by the presence of 3′ BTE, eIF4F and eIF3. These changes can be caused by shifting RNA–protein interactions, or changes to RNA structure caused by those interactions.

### Secondary and tertiary predicted structures

Nearest-neighbor predictions of 5′ UTR and FUS oligomer secondary structure with SHAPE data constraints were calculated using RNAstructure (32) and modeled in RNA2Drawer (33). While changes in SHAPE reactivity can result from restructuring RNA, essentially the same secondary structures are predicted regardless of factor mixtures (Supplementary Figure S1). There are two differences in the structures presented in Supplementary Figure S1b. In the structure without eIFs, the base of SLD is closed by three nucleotide pairs compared to two in the structure with eIFs. This could potentially make this SL slightly more stable in the absence of eIFs. The second change, residues 157 and 244 are paired in the presence of factors and unpaired in their absence. Both of these interactions are predicted with a relatively low level of confidence and as with all predicted structures there may be slight variations even when SHAPE data is used to enhance the reliability of predictions. Neither of these interactions are in areas of eIF interactions. Thus, observed changes in SHAPE reactivity are primarily attributed to RNA-protein binding interactions and changes in long-distance RNA-RNA interactions. Most nucleotides that acquire protection from SHAPE reactivity after the addition of eIF3 are indicative of RNA positions that interact with the eIF3 complex.

To better visualize the shift of eIF3–RNA interactions caused by eIF4F and BTE, predictions of 5′ UTR and FUS oligomer tertiary structures were generated using RNA composer (34,35). Nucleotides protected by eIF3 complexes and their relative distances were visualized in PyMOL. Predicted tertiary structures had the same secondary structures calculated by RNAstructure.

## RESULTS

### SLC binds eIF3 and affects translation efficiency in a largely sequence independent manner

Stem loop C (SLC) of the BYDV 5′ UTR was identified as a potential eIF3 binding site due to sequential (a U-rich hairpin) and structural (an asymmetric internal loop) features that SLC shares with other eIF3-binding structures (Supplementary Figure S2). These SLC-like and eIF3-binding RNA structures help regulate the translation of both cellular (c-JUN) and viral (HCV) mRNAs (24,36–38). To assess the extent to which binding occurred to SLC, a series of oligonucleotide mutants were created. These mutants, as described below were used to assess eIF3 binding to SLC in the absence of other competing structures and to determine the structural and/or sequence features necessary for binding affinity. Earlier studies have shown that eIF3 does not interact non-specifically with similar sized or even larger oligonucleotides (13). In addition, corresponding mutants were created in the SLC of the BlucB_105_ reporter construct to determine the translation of these mRNA.

To assess the significance of a U-rich sequence, the SLC-U mutant was designed to decrease the uracils in the SLC hairpin. SLC-U was inserted into the BlucB_105_ luciferase reporter construct in place of wt SLC (Figure 2A) and translated in WGE. Translation efficiency of BlucB_105_ mutants relative to wt BlucB_105_ are shown in Figure 2B where we see that alteration of the SLC hairpin sequence did not significantly affect the efficiency of luciferase translation (SLC-U: 108 ± 17%).

To assess the translational significance of the SLC internal loop, three mutants that alter or remove the internal loop (SLC-rev, SLC-ILF and SLC-ILR) were inserted into the BlucB_105_ reporter construct (Figure 2A) and translated in WGE. The relative translation efficiencies in Figure 2B show that translation was reduced by the two mutants that altered the SLC internal loop by either reversing the full sequence of SLC (SLC-rev: 68 ± 4%) or by swapping the 5′ and 3′ sides of only the SLC internal loop (SLC-ILF: 51 ± 6%). The greatest translational reduction came from the mutant that removed the internal loop completely (SLC-ILR: 30 ± 4%). Taken together, these results show that the overall structure, and the internal SLC loop in particular, are important for translation. However, sequence appears to be relatively unimportant.

Two additional deletion mutants of BlucB_105_ indirectly highlight the significance of an eIF3-SLC interaction. The 5′ UTR stem loop A (SLA) lacks the potential eIF3-binding features of SLC, and SLA deletion has no effect on WGE translation (dSLA: 96 ± 6%). Because eIF3 simultaneously interacts with 5′ and 3′ UTRs deletion of the 3′ BTE stem loop II (SLII) allows us to look at the translational effect of a potential eIF3 binding site in the 3′ BTE without significantly disrupting known translational requirements of eIF4F-SLI binding and the SLD-SLIII kissing loop (dSLII: 67 ± 11%). Earlier studies have shown that disruption of the “kissing loop” interaction abolishes translational efficiency (9).

The binding affinities of eIF3 for an isolated 36 oligonucleotide of wt SLC and three of the SLC mutants were determined using fluorescence anisotropy (Table 1). Anisotropy binding curves for wt SLC and the SLC-ILR mutant are shown in Figure 2C and D, respectively. The binding curves in the absence of additional factors are similar. The observed almost ten-fold increase in eIF3 affinity for wtSLC and SLC mutants caused by the presence of helicase factors was expected due to the known helicase-dependent binding of eIF3 to the full-length 5′ UTR (13). Comparison of wt and mutant SLC eIF3 binding curves show that only wt SLC sees a relative decrease in binding affinity when eIF4F is added to the helicase factor mix. This is consistent with data described below that show eIF4F aides in repositioning of eIF3 on the 5’ UTR and changes the SLC contacts. The fact that these binding data do not correlate well with the translation data is also consistent with a required repositioning of eIF3 for efficient translation.

**Table 1.** Fluorescence anisotropy binding studies of eIF3 to wt SLC and SLC mutant RNA oligonucleotides in the presence and absence of additional eIFs and ATP as indicated

	**No extra factors**			**eIF4A,B,F and ATP**			**eIF4A,B and ATP**		
**RNA**	***K*_d_ (nM)**	***r*_max –_ *r*_min_**	** $\boldsymbol\chi^{\bf 2}$ **	***K*_d_ (nM)**	***r*_max –_ *r*_min_**	** $\boldsymbol\chi^{\bf 2}$ **	***K*_d_ (nM)**	***r*_max –_ *r*_min_**	** $\boldsymbol\chi^{\bf 2}$ **
SLC wt	43.0 ± 0.9	0.017	0.006	21.3 ± 0.2	0.026	0.002	6.6 ± 0.5	0.016	0.054
SLC-U	35.8 ± 1.4	0.012	0.020	6.9 ± 0.4	0.007	0.022	<5 nM	0.008	0.042
SLC-rev	63.7 ± 1.1	0.025	0.003	<5 nM	0.007	0.070	<5 nM	0.007	0.020
SLC-ILR	56.3 ± 2.4	0.020	0.021	<5 nM	0.010	0.043	<5 nM	0.010	0.039

### Differential SHAPE footprinting shows addition of eIF4F affects 5′ UTR-eIF3 interaction on SLC

**Figure 3.**
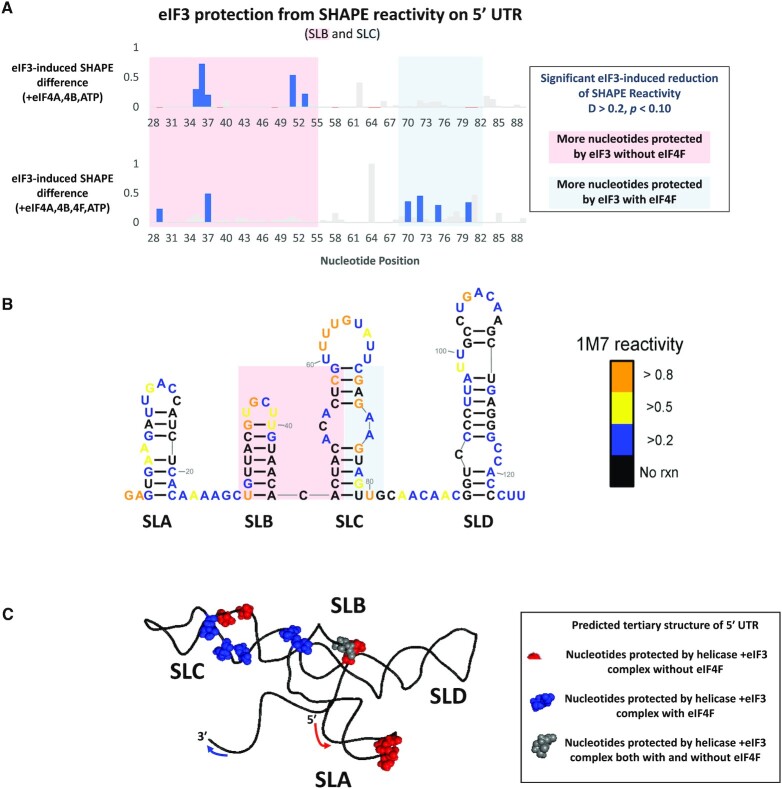
(**A**) Nucleotides of 5′ UTR RNA oligo that were protected by eIF3 in the presence of eIF3 and helicase factors. The upper plot and lower plots lack and include eIF4F in their helicase factor mix, respectively. (**B**) SHAPE data of the 5′ UTR RNA oligo with red and blue boxes highlighting areas that have better eIF3 protection in the presence (blue) or absence (red) of eIF4F. (**C**) predicted tertiary structure of 5′ UTR RNA oligo showing the predicted proximity of the eIF3-protected nucleotides.

Figure 3A shows nucleotide positions on the 5′ UTR RNA oligomer that are protected from SHAPE reactivity by an eIF3 complex. The plots of Figure 3A were generated by comparing SHAPE data collected both with and without eIF3 present (Supplementary Figure S3). The blue bars in the Figure 3A indicate nucleotide positions that saw statistically significant drops in reactivity when eIF3 was present. Significance is defined as having an eIF3-induced drop in SHAPE-reactivity >0.2 and a *P*-value <0.1. The upper and lower plots show the differing eIF3 binding positions when eIF4F is excluded or included, respectively.

The eIF3 complex preferentially interacts with different nucleotides depending on whether eIF4F is included in the complex. In the absence of eIF4F, the eIF3 complex protects SLB and the 5′ side of the SLC internal loop (SLC-IL) from SHAPE reactivity. In the presence of eIF4F, the eIF3 complex maintains some protection of SLB, loses protection of the 5′ SLC-IL, but gains protection on the 3′ SLC-IL. This ability of eIF4F to direct eIF3 interactions with 5′ UTR nucleotides is highlighted by red and blue boxes in Figure 3A and B. It is important to note that addition of eIF4F to the eIF3-5’ UTR not only protects additional nucleotides (e.g. residues 70, 72, 75 and 80) as would be expected with a larger protein complex, but also eIF4F exposes nucleotides previously protected by eIF3 (e.g. residues 51 and 53) suggesting movement of the eIF3 on the 5’ UTR. Figure 3B shows the sequence, secondary structure, and SHAPE reactivity of the 5′ UTR RNA oligomer allowing for better visualization of eIF3 binding sites highlighted by the red and blue boxes and emphasizing the change in eIF3 interactions across the SLC-IL. A more detailed plot of 5′ UTR SHAPE reactivity is shown in Supplementary Figure S4A.

Figure 3C enhances visualization of eIF3-binding further by showing a predicted tertiary structure of the 5′ UTR RNA oligomer built from the SHAPE-constrained secondary structure in Figure 3B. The predicted proximity of protected nucleotides on SLB and SLC could explain how eIF3-SLB interactions can be partially maintained (grey spheres) while eIF3-SLC interactions shift after addition of eIF4F (red and blue spheres). It is interesting to note the observed shift across SLC-IL and compare with the IVT of SLC-IL mutants shown in Figure 2B. Given that binding of eIF3 in the absence of eIF4F is at the 5’ side of SL C and in the presence of eIF4F at the 3’ side of SLC with little protection of the apical loop in either case, it is not surprising that mutants SLC U and SLC rev had little effect on translation. However, abolishing the internal loop which is protected on one side in the absence of eIF4F and on the opposite in the presence of eIF4F had a significant effect on translation consistent with this region of the 5’ UTR being important for binding and translation.

### BTE alone as well as BTE and eIF4F alter eIF3 binding

The ability of eIF3 to bind both the 5′ and 3′ UTR of BYDV specifically, simultaneously, non-competitively and in a helicase-dependent manner was identified by analyzing BYDV UTRs both individually and together in trans (13). To better characterize 5′ and 3′ UTR interactions, and the simultaneous interactions of both UTRs with eIF3, a more physiological condition, we designed a new oligomer containing both UTRs in cis connected by a 12-nucleotide linker sequence. SHAPE, SHAPE footprinting, and binding studies in Supplementary Figure S4 show that this fusion oligomer containing both UTRs (FUS) maintains most expected characteristics of the individual UTRs including expected 5′ and 3′ UTR structures, an intact kissing loop, and eIF4F binding to BTE-SLI (12,39). This increases our confidence that other observed eIF-FUS interactions from differential SHAPE footprinting experiments hold translational significance.

**Figure 4.**
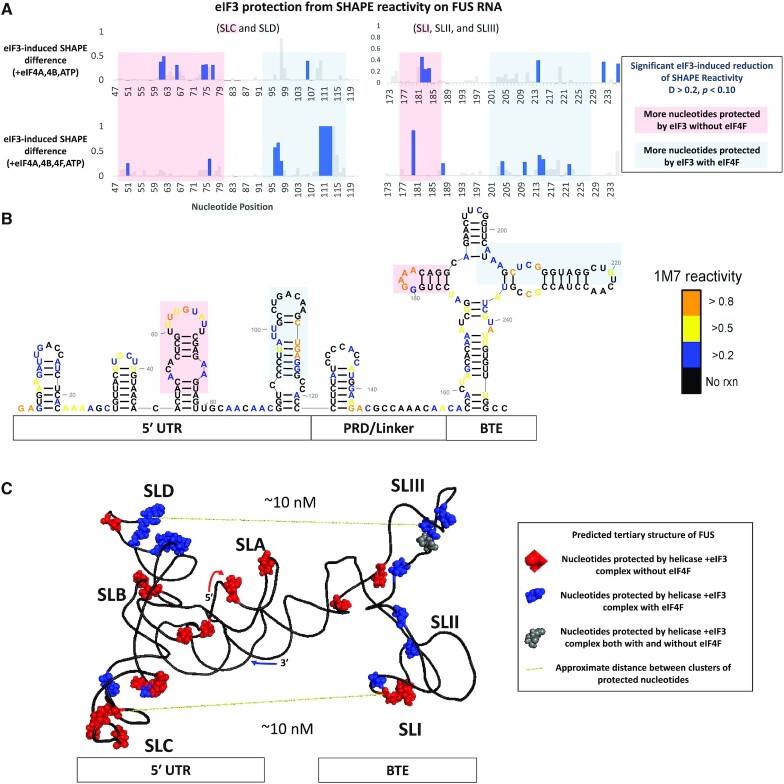
Nucleotides of FUS RNA oligo that were protected by eIF3 in the presence of eIF3 and helicase factors. (**A**) SHAPE reactivity of FUS RNA protected by eIF3 in the absence (upper plot) and presence (lower plot) of eIF4F in the helicase factor mix, respectively. (**B**) SHAPE data of the FUS RNA oligo with red and blue boxes highlighting areas that have better eIF3 protection in the presence (blue) or absence (red) of eIF4F. (**C**) tertiary structure prediction of 5′ UTR RNA oligo showing the predicted proximity of the eIF3-protected nucleotides. It should be noted that long range interactions are not well predicted by modeling and there is likely flexibility in the linker between the 5’ and 3’ UTRS.

Figure 4 shows that in the presence of BTE and helicase factors without eIF4F, eIF3 will interact with the 5′ UTR SLC hairpin. While this interaction with SLC also occurred in the absence of BTE, SLC is much more protected than when eIF3 interacted with only the 5’ UTR. This may partially explain why some mutations of SLC had a significant effect on translation, but a much smaller effect on binding to the 5’ UTR. The protein may be able to bind to SLC mutants, but be unable to make contacts necessary for reorientation. In addition, SLI of the BTE is also protected in the absence of eIF4F suggesting possible alignment for the kissing loop interaction. While eIF4F was able to shift eIF3 more toward the 3’ side of SLC in the absence of BTE, the protein was not shifted to SLD as is the case when BTE is present. Because the FUS oligo contains BTE in cis we can also analyze the eIF4F-induced changes to the eIF3 footprint on BTE that correspond with the shifts on the 5′ UTR. Figure 4 shows a loss of eIF3 protection on the hairpin of SLI and significantly increased eIF3 protection on SLIII when eIF4F is present. These eIF4F-induced shifts of eIF3 protection on both UTRs show movement towards the two RNA loops involved in the kissing-loop interaction (5′-SLD and 3′-SLIII). Figure 4C shows that the structures in 5′ and 3′ UTR that are bridged by the eIF3 complex both without eIF4F (SLC and SLI) and with eIF4F (SLD and SLIII) are roughly equidistant in the predicted tertiary model. These predicted ∼10 nm distances are well within the reach of the large, 800 kDa eIF3 complex (40,41). It should be noted, however, that these SL interactions must be dynamic and transient in order to transfer eIFs and ribosomes from the 3’ BTE where they are recruited (9–12,39) to the 5’ UTR where the SL interactions at some point must be disrupted to allow ribosomal scanning.

In order to try to gain further insight into the orientation of eIF3 on the FUS oligomer, additional binding and SHAPE footprinting studies were carried out with the eIF3a N-terminal domain and eIF3b subunits (Supplementary Figure S5). The N-terminal domain of eIF3a binds the 5′ UTR and protects SLC in a helicase dependent manner. While eIF3 shifts from SLC to SLD in the presence of eIF4F, the eIF3a subunit is bound to SLC even in the presence of eIF4F and does not shift to SLD. This suggests, not surprisingly, that additional subunits are necessary for full interaction and function of eIF3a. It also suggests the location of eIF3 prior to the shift induced by eIF4F. Binding studies of eIF3b show that it will bind 3′ BTE (with relatively low affinity) but not 5′ UTR when no additional factors are present. When helicase factors including eIF4F are present, eIF3b only binds to the 5′ UTR.

## DISCUSSION

The translation of barley yellow dwarf virus relies on its 3’ translational enhancer, BTE, to recruit translational machinery to the 3’ BTE, communicate with its 5’ UTR, and transfer said machinery onto its 5’ UTR. The mechanism of this complex process, however is not well understood. In this study, we explore the 3’ BTE and 5’ UTR binding sites of eIF3 and how this binding changes with addition of key eIFs. The results of this study build on previous work to establish an expanded model of BYDV translation in which eIF4F promotes the rearrangement of eIF3 binding on the 5’ UTR suggesting new roles for both factors.

BTE contains a conserved 17 nucleotide sequence (bases 4833–4849 in wild type BYDV and 172–188 in the FUS oligomer shown in Figure 4B). This 17-base sequence contains BTE SLI and is found on all BYDV-like 3’ CITEs (8). Our data show that eIF3 protects SLI from SHAPE modification in the presence of active helicase factors eIF4A and eIF4B. This includes segments of SLI that have been shown to bind eIF4F using similar SHAPE footprinting techniques (11,12,39) (Supplementary Figure S4C). As shown in Figure 4 and Supplementary Figure S6, the SHAPE footprint of the eIF3 complex that includes eIF4F shows fewer SLI nucleotides protected than the complex without eIF4F. This indicates that eIF3 and eIF4F are both shifting away from this stem loop and points to the eIF3 complex shift as a potential means of transferring BTE-bound factors to the 5’ UTR.

This shift of factors can fit with results that identify an 18S rRNA-complimentary sequence of 6 bases (GAUCCU) in the conserved sequence as a potential binding site for the 40S ribosomal subunit. Active helicase factors eIF4A, eIF4B and eIF4F expose bases in that complementary sequence that are otherwise involved in secondary structure which helps recruit 40S ribosomes to BTE with *K*_d_ = 120 nM affinity (12). When eIF3 is added to those helicase factors the resulting complex appears to move away from SLI and this potential 40S binding site. This movement of factors could be connected to the stronger 40S-BTE binding (K_d_ < 5 nM) measured when both eIF3 and eIF4F are present (13). This enhanced 40S binding is comparable to the affinity of 40S ribosomes for eIF4F bound to the m^7^GTP cap in canonical, cap-dependent translation (42) and suggests a correlation between the observed shift of the eIF3 complex and the previously reported, cap-like role of BTE in recruiting factors for BYDV translation (19,43,44). The translational necessity of this eIF3–eIF4F interaction and the resulting shift away from SLI is further supported by the presence of an eIF3 binding domain in the minimal, translationally active core of eIF4G (the BTE-binding component of eIF4F) in BYDV translation (45).

The binding and translation studies shown in Figure 2 explore the role of SLC in recruiting factors to the 5′ UTR and the importance of the proposed pre-shifted eIF3 bridge between SLC and SLI. The lack of any SLC-U effect on binding or translation suggests that SLC primary sequence is of little consequence provided secondary and tertiary structures are maintained. The SLC-rev and SLC-ILF mutants were designed to maintain the secondary structure of SLC but, due to the slightly asymmetrical internal loop, alter the position of the SLC hairpin relative to SLI and disrupt the eIF3 bridge between these loops. Similarly, SLC-ILR removed the internal loop to disrupt both the orientation of SLC and remove the internal loop as a structure that eIF3 might recognize. The strong eIF3 affinity for SLC-rev and SLC-ILR coupled with the weak translational efficiency of BlucB_105_ containing SLC-rev, SLC-ILF and SLC-ILR suggests that the formation of a proper SLC-SLI bridge or the ability of eIF3 to shift away from it was effectively disrupted by these mutants. This supports the existence and physiological relevance of a pre-shifted eIF3 bridge. BYDV translation efficiency assays performed in W. Allen Miller's lab that removed SLC have also shown SLC can affect translation especially when the assays are carried out in protoplasts (9). This aligns with the reduction of efficiency caused by SLC-ILF and SLC-ILR mutants in WGE that dropped translation to 51% and 30%, respectively. This suggests that the proper structure and orientation of the SLC-IL is central to the translational role of SLC. The dSLII reduction to 67% translational efficiency and the observed eIF3 protection at the 5’ base of SLII (nucleotide 204) after the addition of eIF4F (Figure 4) suggests that SLII could have a minor role in the eIF3 shifting process happening on the 3′ side of the eIF3-UTR bridge. It is important to note that no SLC mutants studied or referenced in this report fully abolished translation. Thus, these data suggest that binding factors on SLC and the shift of factors away from SLC and SLI plays a role in enhancing the efficiency of BYDV translation, but it does not prevent BYDV from utilizing alternate, less efficient mechanisms of translation.

**Figure 5.**
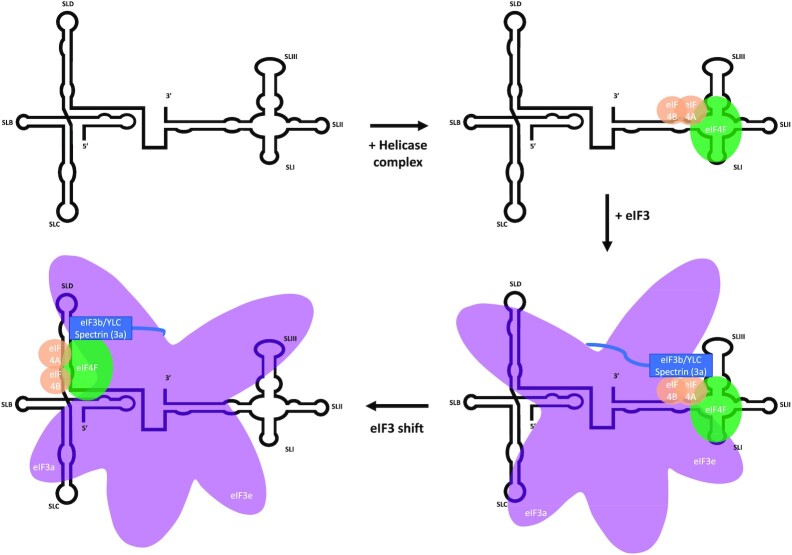
A proposed model for eIF3-eIF4F-UTR interactions during BYDV translation initiation. The eIF3 core maintains its bridging of the two UTRs while the reorientation of eIF3 peripheral subunits and eIF4F is triggered by an eIF4F/eIF3 interaction on the 3′ BTE. This rearranging of eIF3 enables the 40S ribosome to more efficiently assume a translationally active configuration on the 5 ′ UTR.

We propose that the ability of eIF3 to facilitate greater translation efficiency in BYDV is related to the known ability of eIF3 peripheral subunits to undergo significant structural rearrangements during translation initiation (46,47). Supplementary Figure S5 shows how the peripheral subunit eIF3b only binds BYDV 5′ UTR in the presence of helicase factors and only binds BYDV 3′ BTE in the absence of helicase factors. Subunit eIF3b has a known interaction with eIF4B (48), one of the helicase factors necessary to facilitate eIF3 binding to both BYDV UTRs (13). This points to a role for eIF3b and other peripheral subunits in the yeast like core (YLC) subcomplex of eIF3 (48,49) in bringing BTE-bound eIFs to the 5′ UTR where they are needed for translation (Figure 5). The YLC is attached to core subunits of eIF3 via the flexible spectrin domain found at the C-terminus of eIF3a, a core subunit of eIF3 (41). The 5-pronged structure of the eIF3 core subunits are anthropomorphized as arms, legs, and a head and represented by the pink structure in Figure 5. The footprint of the N-terminal domain of eIF3a (left arm of the eIF3 core complex) on the FUS oligo shows a binding site on the hairpin of SLC (Supplementary Figure S5). This SLC protection and a known interaction between subunit eIF3e (right arm of the eIF3 core complex) and eIF4F (50,51) allows us to orient the eIF3 complex on the FUS oligomer in its pre-shifted state between 5′ SLC and 3′ SLI.

This 3’ UTR and eIF3 dependent enhancement of BYDV translation is reminiscent of some translation mechanisms employed by viral mRNAs with internal ribosomal entry site (IRES). The hepatitis C virus and classical swine fever virus both contain 3’ cis-acting structures that significantly increase translation by interacting with both eIF3 and the 40S ribosome (15,52,53). Our expanded understanding of the role of eIF3 in this BYDV mechanism may prove useful in characterizing a plethora of plant virus with similar structural elements. Red clover necrotic mosaic virus is a prime example of this as it contains both a BYDV-like translation element in the 3’ UTR and a SLC-like structure (labeled SLIII) in the 5’ UTR of its RNA1 (54).

In conclusion, this study supports an expansion of a translation initiation model for genomic BYDV mRNA and introduces a novel role for eIF4F in reconfiguring the initiation complex of eIF3 to help shuttle factors from 3’ BTE to the 5’ UTR (Figure 5). This shuttling and reconfiguring process is triggered by eIF3 interactions with BTE and eIF4F. Furthermore, this model adds to the repertoire of eIF3 functionality and to our understanding of viral RNA ingenuity by highlighting the ability of these highly structured UTRs to use eIF4F and eIF3 in this novel manner.

## DATA AVAILABILITY

RNAstructure is available from the Matthews lab webpage (https://rna.urmc.rochester.edu/RNAstructure.html).

RNA2drawer is available in the SourceForge repository (https://sourceforge.net/projects/rna2drawer/).

RNA composer is available at (http://rnacomposer.cs.put.poznan.pl/).

CEQ programs are available on the Laederach Lab webpage (https://ribosnitch.bio.unc.edu/software/).

SHAPEfinder software is available in the GitHub repository (https://github.com/drsuuzzz/ShapeFinder).

PyMOL software is available at (https://pymol.org/2/).

## Supplementary Material

gkac284_Supplemental_File
