# Supplementary material for: Eukaryotic initiation factor 4F promotes a reorientation of eukaryotic initiation factor 3 binding on the 5′ and the 3′ UTRs of barley yellow dwarf virus mRNA
Source: Nucleic Acids Res. 2022 Apr 21;50(9):4988–99. doi: 10.1093/nar/gkac284 (PMC9122605; doi:10.1093/nar/gkac284)
Supplement: gkac284_Supplemental_File [file gkac284_supplemental_file.pdf]

Supplemental Table 1. Sequences and uses of short RNA oligomers with highlights showing significant regions of SHAPE RNAs

Experiment	Oligomer Name	Sequence
Binding	SLC-WT oligo	GCACUACACACUCGUUUUGUAUUCGAGAAGUAGUGC
Binding	SLC-U	GCACUACACACTCGUAAAGAAUUCGAGAAGUAGUGC
Binding	SLC-Rev	CGUGAUGAAGAGCUUAUGUUUUGCUCACACAUCACG
Binding	SLC-ILR	GCACUACACACUCGUUUUGUAUUCGAGTGTGUAGUGC
SHAPE	5′ UTR	GAGUGAAGAUUGACCAUCUCACAAAAGCUGUUACGUGCUUGUAACACACUACACACUCGUUU<br>UGUAUUCGAGAAGUAGUUGCAACAACGGUCCCCUUAUUGCCUGACAAGCUGAGGGCCACCCU<br>UCUAUCCCCACCGCGCGCAUGGAAGACGCCAAAAACAUAAAGAAAGGCCCGGCGCCAUUCUAU<br>CCUCUAGAGGAUGGAACCGCUGGAGAGCAACUG
SHAPE	FUS	GAGUGAAGAUUGACCAUCUCACAAAAGCUGUUACGUGCUUGUAACACACUACACACUCGUUU<br>UGUAUUCGAGAAGUAGUUGCAACAACGGUCCCCUUAUUGCCUGACAAGCUGAGGGCCACCCU<br>UCUAUCCCCACCAUGGAAGACGCCAAACAACACCACUAGCACAAAUCGGAUCCUGGGAAACAG<br>GCAGAACUUCGGUUCAUAAGCUCGGGUAGGCUGUCAACCUACCGCCGUAUCGUAUUGUGUU<br>UGGCCGCCAUUCUAUCCUCUAGAGGAUGGAACCGCUGGAGAGCAACUG

Key
5′ UTR
3′ BTE
luciferase

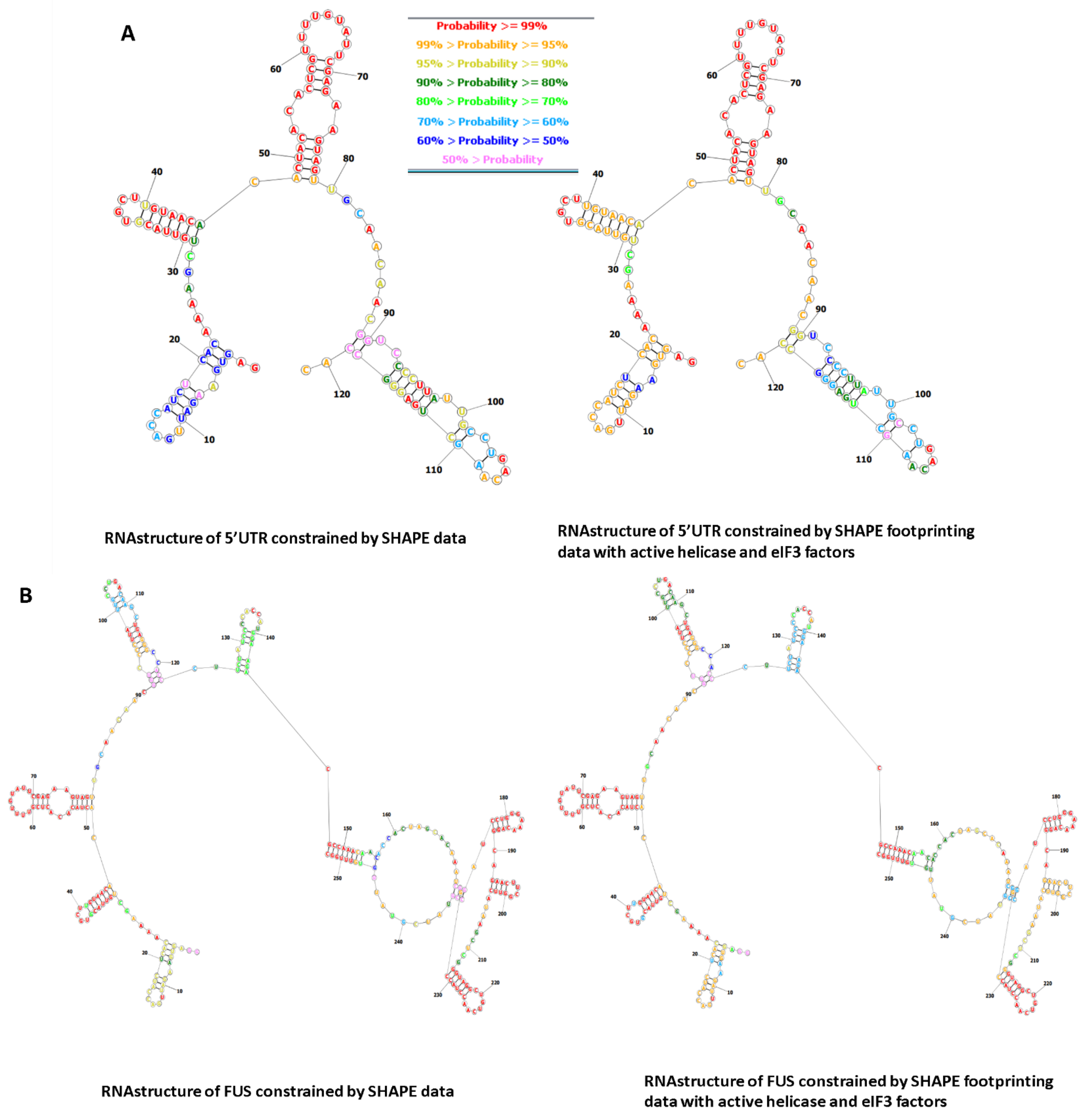

Supplemental Figure 1. (A) Maximum expected accuracy (MEA) structure prediction from RNAstructure shows consistent prediction of 5' UTR secondary structures with high confidence in secondary structural elements regardless of whether helicase and eIF3 factors are present. (B) The same phenomenon is observed for the FUS oligomer structural prediction. Some of the integrity of SLIV is lost in both conditions likely due to structural flexing caused by RNA-RNA interactions across the UTRs, however SLI, SLII, and SLIII are well defined.

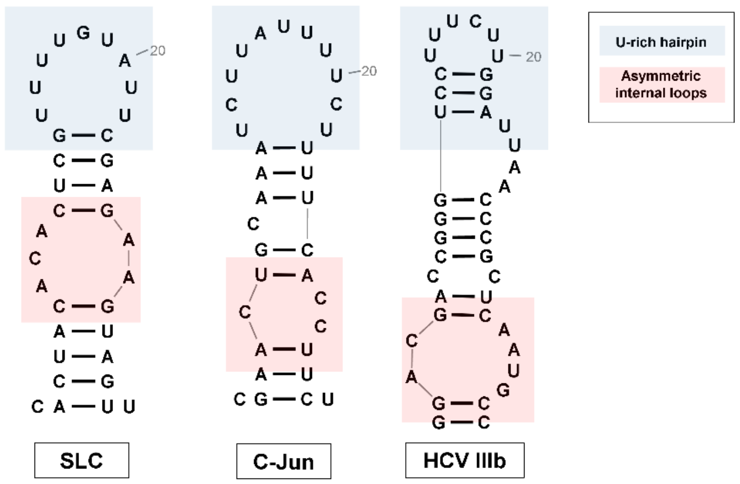

Supplemental Figure 2. Structural and sequence comparison of the 5' SLC structure of BYDV with two other known eIF3-binding structures from the internal ribosomal entry sites from a cellular mRNA (C-jun) and a viral mRNA (HCV).

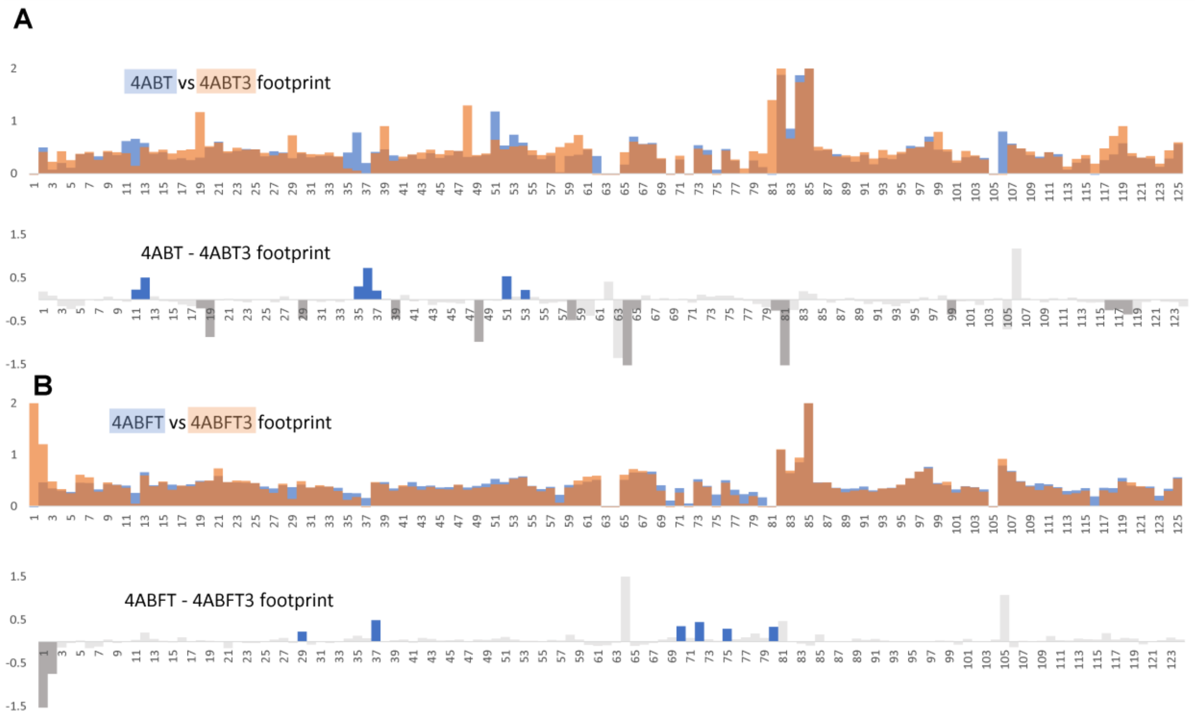

Supplemental Figure 3. (A) the SHAPE data used to generate the upper plot of figure 3A (B) the SHAPE data used to generate the lower plot of figure 3A. Initiation factor mixes used to generate each plot are represented with abbreviations: 4ABT is eIF4A, eIF4B, and ATP; 4ABFT is 4ABT plus eIF4F; 4ABT3 and 4ABFT3 are, respectively, 4ABT and 4ABFT both with eIF3 included. The lower plots are the difference between SHAPE footprinting data excluding and including eIF3.

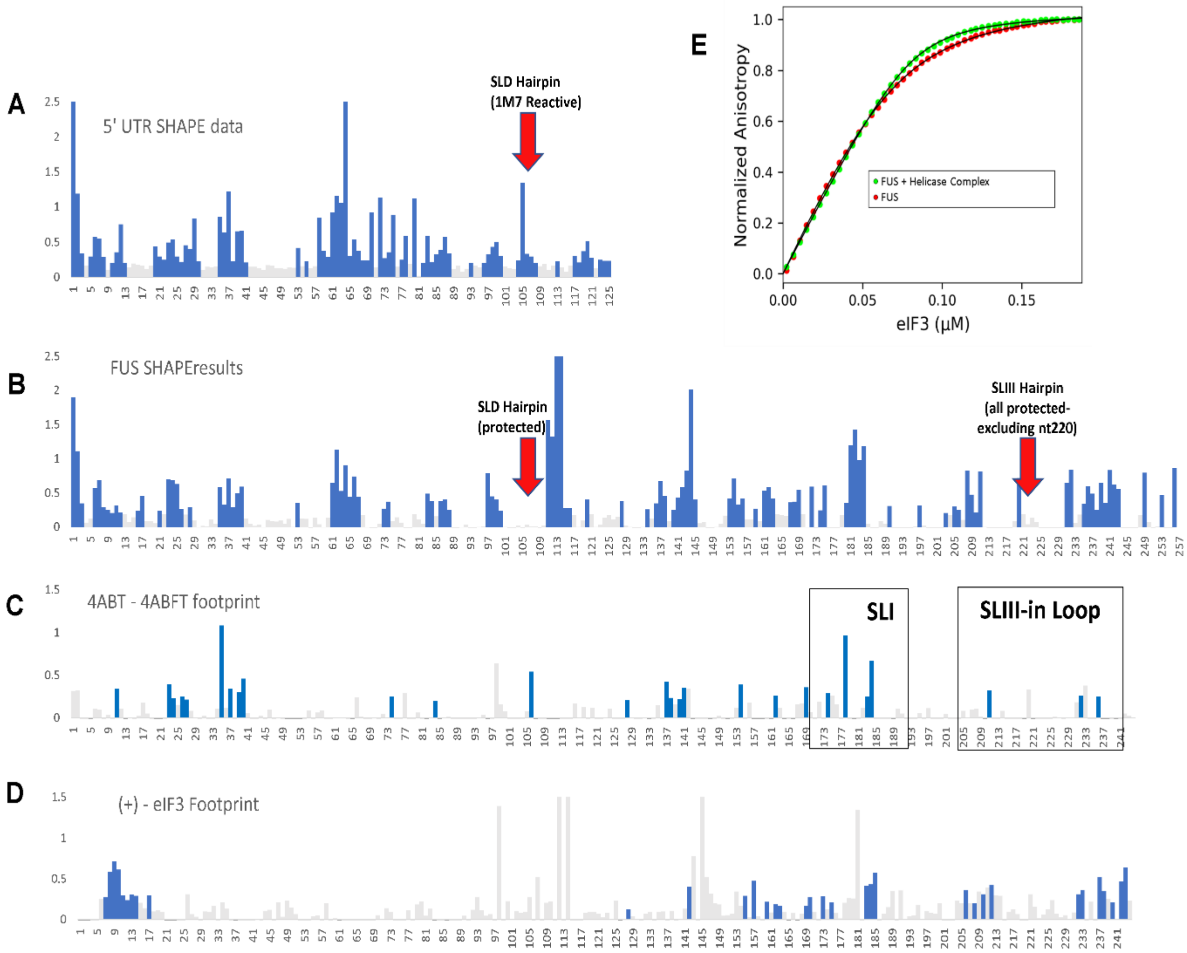

Supplemental Figure 4. (A) the SHAPE data showing the structure of the 5′ UTR with the SHAPE reactive kissing loop interaction emphasized with a red arrow. All positions with SHAPE reactivity greater than 0.2 are marked blue. (B) the SHAPE data showing the structure of FUS RNA with the non-reactive nucleotides of SLD and SLIII highlighted with red arrows. Blue positions marked as in (A). (C) the eIF4F footprint showing protection on SLI and the SLIII-internal loop (D) the footprint of eif3 without any additional helicase factors. No affinity for 5′ UTR as expected, some affinity for BTE maybe due to increased flexibility of SLIV. (E) Green data show binding of eIF3 to FUS in the presence of helicase factors (K_d = 26.3 ± 2.5 nM, r_{max} = 0.142, χ^2 = 0.054). Red data show eIF3 binding without any factors or ATP (K_d = 27.6 ± 1.4 nM, r_{max} = 0.156, χ^2 = 0.014).

A

		Subunit binding affinities: K_ds ± SD (nM)					
		eIF3a			eIF3b		
RNA	Additional Factors	K_ds (nM)	Δr	χ^2	K_ds ± SD (nM)	Δr	χ^2
5′ UTR	none	440 ± 39	0.011	0.103	NB	NB	NB
	eIF4A, B, F, ATP	187 ± 14	0.008	0.111	21 ± 2	0.007	0.024
3′ BTE	none	237 ± 12	0.015	0.014	443 ± 41	0.020	0.037
	eIF4A, B, F, ATP	61 ± 4	0.019	0.020	NB	NB	NB

B

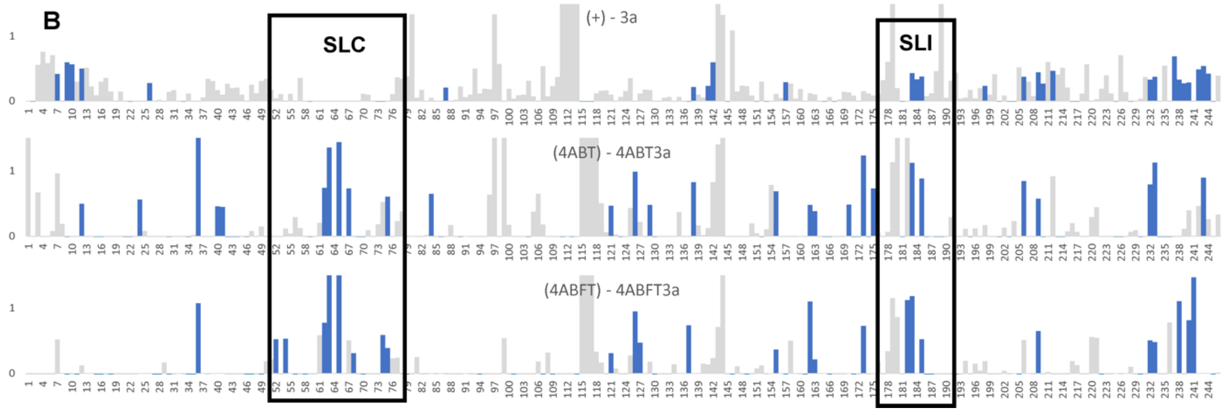

Supplemental Figure 5. (A) Binding results for eIF3a and eIF3b subunits interacting with the 5′ UTR and 3′ BTE show eIF3a interacts with both UTRs regardless of helicase factors while eIF3b interacts selectively with one of the UTRs depending on the presence of helicase factors. NB stands for non-binding. (B) eIF3a SHAPE footprinting show that subunit 3a requires helicase factors either with or without eIF4f to interact with SLC. The eIF3a subunit does not undergo an eIF4F-induced shift without the rest of eIF3 present.

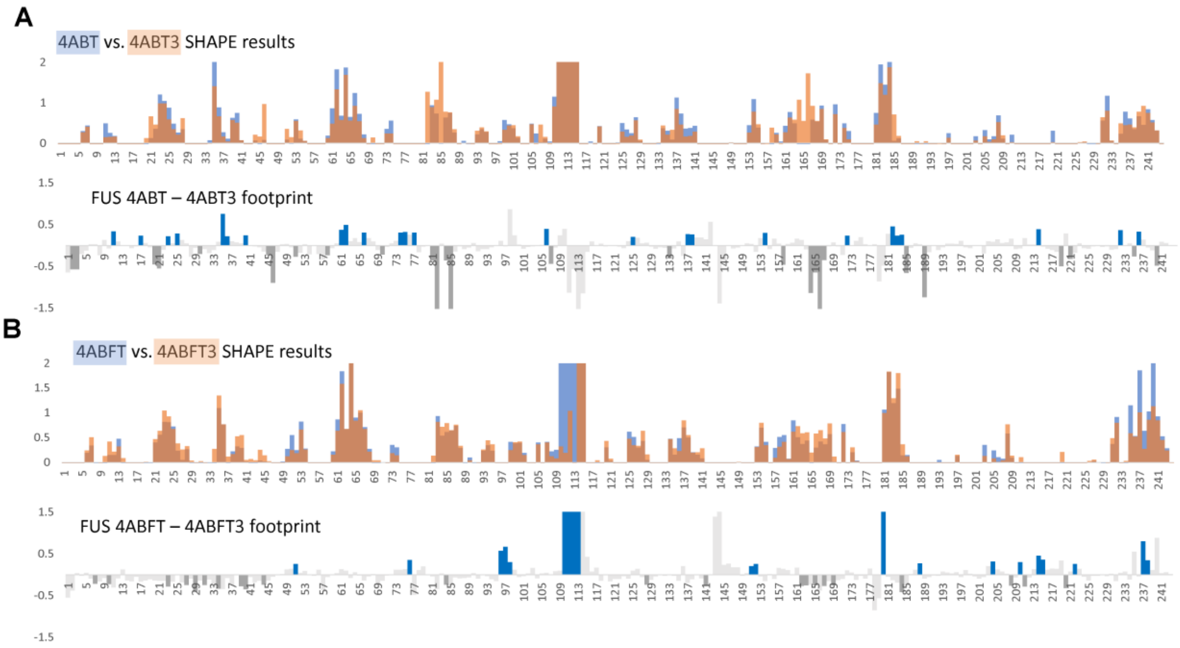

Supplemental Figure 6. (A) the SHAPE data used to generate the upper plot of figure 4A (B) the SHAPE data used to generate the lower plot of figure 4A. Initiation factor mixes used to generate each plot are represented with abbreviations: 4ABT is eIF4A, eIF4B, and ATP; 4ABFT is 4ABT plus eIF4F; 4ABT3 and 4ABFT3 are, respectively, 4ABT and 4ABFT both with eIF3 included. The lower plots are the difference between SHAPE footprinting data excluding and including eIF3. The highlighted nucleotides in Figure 4C only show changes in SHAPE reactivity on the UTR structures themselves, the bottom plots of this supplemental figure show additional bases in the regions between the main UTR structures that had significant, but less pronounced changes in eIF3 SHAPE reactivity protection (nt 125, 137, 138, 151-153).
